# Predicting the Sustained Implementation of an Evidence-Based Parenting Program: A Structural Equation Modelling Approach

**DOI:** 10.1007/s10488-022-01226-x

**Published:** 2022-11-05

**Authors:** Tianyi Ma, Cassandra L. Tellegen, Jenna McWilliam, Matthew R. Sanders

**Affiliations:** 1 Parenting and Family Support Centre, The University of Queensland, 13 Upland Road, 4072 St Lucia, Australia; 2 Triple P International, Brisbane, Australia

**Keywords:** Evidence-Based Program, Implementation, Sustainability, Parenting, Triple P

## Abstract

**Purpose:**

Sustained implementation is required for evidence-based parenting programs to promote children and their families’ wellbeing at the societal level. Previous literature has examined the role of a range of different factors in enhancing sustainability. However, the inter-relationship between, and the relative importance of different factors remain largely unknown. The overall aim of this study is to identify predictors of sustained program use, the relative importance of factors, and potential mediation pathways.

**Methods:**

We surveyed 1202 practitioners who were trained in at least one variant of the Triple P-Positive Parenting Program, at least one and half years before data collection. The present data were linked with data collected during professional training. We first examined the independent effect of each factor on sustained program use, then, developed and evaluated a structural equation model of sustained program use.

**Results:**

The structural equation model explained a considerable amount of variance in sustained program use, with seven positive predictors and one negative predictor identified. Organisational support was identified as a key facilitator, which was not only positively linked with other facilitators but also had an independent positive effect. Perceived usefulness of the program was the most important practitioner-level facilitator, which might be contributed by both research-based evidence and practice-based evidence. Practitioners’ self-regulation in program delivery impacted sustained use by influencing other factors such as perceived usefulness of the program.

**Conclusion:**

The findings provided insight into factors influencing the sustainability of evidence-based parenting programs which could be used to inform future implementation practice.

**Supplementary Information:**

The online version contains supplementary material available at 10.1007/s10488-022-01226-x.

## Introduction

The use of evidence-based parenting programs has the potential to reduce the prevalence of mental health disorders in, promote the resilience for, and foster the healthy development of children (Sanders, 2012; Scott, 2010). At the same time, parents tend to benefit from participating in evidence-based parenting programs as well, with outcomes including improved parenting practices, decreased distress, and less conflict with partners (Sanders et al., 2014). The Triple P – Positive Parenting Program is one system of evidence-based parenting programs which has a strong evidence base and wide international reach (Sanders, 2012; Sanders et al., 2014). When taking a public health approach in the dissemination of Triple P, positive outcomes including reductions in child maltreatment rates have been shown to be achieved at a population level (Prinz et al., 2009).

Despite the benefits evidence-based parenting programs might have on child and parent wellbeing, only a small proportion of parents have participated in them globally (Prinz & Sanders, 2007). Having a large, multidisciplinary workforce to provide a range of services available for parents could promote the accessibility of evidence-based parenting programs to help more families (Prinz & Sanders, 2007). The ability to maintain this workforce to deliver the program with fidelity at sufficient intensity and continuously in an effort to achieve anticipated goals and population outcomes is called program sustainability (Moore et al., 2017; Shelton et al., 2018; Shelton & Lee, 2019). The challenge of ensuring the sustainability of evidence-based public health programs including parenting programs has received increasing scholarly attention.

Multiple theoretical models and conceptual frameworks have been developed to predict, describe, and explain the successful implementation and sustainment of evidence-based public health programs. For example, Shelton et al., (2018) reviewed sustainability literature and suggested the different factors can be conceptually categorised into five interconnected dimensions. Besides the broader socio-political environment, the four dimensions include organisation (e.g. resources, program champions, and support), process (e.g. training, supervision, and partnership), intervention characteristics (e.g. adaptability, fit with context and population, and effectiveness), and implementer characteristics (e.g. skills, attitude, and motivations). These dimensions are generally consistent across different implementation frameworks (e.g. Aarons et al., 2011; Damschroder et al., 2009; Stirman et al., 2012).

However, due to the absence of psychometrically sound measurements, the five-dimension categorisation has mainly been theory-driven and based on reviewing prior literature, without being comprehensively evaluated (Shelton et al., 2018; Shelton & Lee, 2019). Not all dimensions are well established (Shelton et al., 2018), and different frameworks tend to emphasise different key factors over the others (Scheirer & Dearing, 2011). Besides evaluating implementation frameworks at the framework level, more empirical sustainability studies are needed to fill in remaining research gaps at the individual factor level about the relative importance of factors and the interrelationships among different factors (Scheirer & Dearing, 2011; Shelton et al., 2018; Stirman et al., 2012).

### Self-Efficacy and Self-Regulation

One key factor of program sustainability is practitioners’ self-efficacy in program delivery, which is defined as a practitioner’s belief in their capabilities to achieve the desired outcomes such as changing the parenting behaviours of program participants (Bandura, 1977; Sanders et al., 2019). Self-efficacy or similar concepts have been included in multiple sustainability or implementation frameworks as a practitioner/implementer-level factor since its role in predicting program use has been demonstrated in many studies (e.g. Damschroder et al., 2009; Shelton et al., 2018). Practitioners who have higher levels of self-efficacy are anticipated to be more likely to initiate program use and to display higher levels of persistence in the face of obstacles.

In the area of parenting intervention research, specifically in studies using Triple P, higher self-efficacy has been generally linked with higher levels of program use. The association between self-efficacy and program use is likely to be bi-directional. Practitioners who feel more confident in delivery would be more willing to use the program, while more experience with offering the program would improve self-efficacy. For example, after two years of implementation, highly efficacious practitioners tended to report more frequent delivery of Triple P (Charest & Gagne, 2019). Preliminary evidence suggests that self-efficacy partially mediates the impact of a range of other factors such as organisational support, ease of delivery, and program support on sustained program use (McWilliam, 2016; Turner et al., 2011). At the same time, practitioners’ self-efficacy tended to increase over time through using Triple P with strong implementation processes and sufficient organisational support (Côté & Gagné, 2020a, b). A qualitative study confirmed these findings, where practitioners identified self-efficacy as a main contributor to their sustained Triple P delivery (Shapiro et al., 2015).

Arguably, a more comprehensive approach to investigate self-efficacy would be to investigate it within the self-regulatory framework. Self-regulation is defined by Karoly (1993, p. 5) as “those processes … that enable an individual to guide his/her goal-directed activities over time and across changing circumstances (contexts)”. For parenting practitioners, it refers to having the ability to self-monitor, to adapt their own attribution and behaviour, as well as to be independent problem-solvers (Sanders & Mazzucchelli, 2013; Sanders et al., 2019). Self-regulation is proposed to have five underlying key elements: besides self-efficacy, other key elements are self-sufficiency - having enough knowledge and skills to deliver the program; self-management - having the skills to improve their program delivery; personal agency - attributing positive changes to their own rather than chance or contextual events; and problem-solving- ability to develop and execute a plan to solve a defined problem (Sanders & Mazzucchelli, 2013; Sanders et al., 2019).

Self-regulation is one of the core principles embedded in the development of the Triple P program (McWilliam et al., 2016; Sanders & Mazzucchelli, 2013). All participants, from parents to practitioners, are expected to strengthen their self-regulation over the course of participation. Therefore, Triple P practitioner training has adopted an active skills training approach that emphasises self-directed learning, personal goal setting for skill development, self-evaluation, and problem-solving (Turner & Sanders, 2006). During consultations, rather than providing specific answers to parents’ concerns, practitioners are required to guide parents to promote parental self-regulation in raising their own child with a range of strategies including modelling. Thus, given its potential use in predicting sustained implementation and its significance in Triple P, it is important to uncover the relationship between program providers’ self-regulation and their sustained program use.

### Organisational, Program-Related and Other Practitioner Factors

Besides practitioners’ self-regulation capacity, other individual level factors may also affect sustained program use. Shapiro et al., (2015) found that, during interviews, Triple P practitioners identified the effectiveness of the program as the most important reason for sustained delivery. Observing positive changes in clients’ families and even their own families motivated them to continue offering the program. This ability to produce effective changes is closely related to self-regulation, where practitioners with higher self-regulation are more likely to receive positive feedback and attribute success to their delivery (Sanders & Turner, 2005). A barrier to sustainability is program interference, where practitioners who thought that Triple P delivery affected their personal life or clashed with their preferred theoretical approach tended to use Triple P less over time (Sanders et al., 2009; Shapiro et al., 2015). Practitioners’ personal values and attitudes might also affect sustained program use, and these factors have also been commonly included in implementation frameworks (Aarons et al., 2011; Damschroder et al., 2009; Shelton et al., 2018).

Many practitioners work within service organisations, and organisational factors play a major role in implementation. Consistent with sustainability frameworks, sustained Triple P use was predicted by practitioners’ perceptions about whether their organisations are supportive of using the program (Asgary-Eden & Lee, 2012; Hodge et al., 2017; Seng et al., 2006). Recognising the importance of this, Triple P’s program purveyor organisation Triple P International developed the Triple P Implementation Framework. Using this, Implementation Consultations provide support across multiple implementation phases, from early engagement to maintenance to build the implementation capacity of implementing organisations (McWilliam et al., 2016). Supportive organisations would have adequate physical environments, allocate sufficient funding and resources, prioritise program delivery, facilitate workplace support and recognition, as well as provide quality supervision. With sufficient organisational support, practitioners who held negative initial views toward the implementation decision tended to develop more favourable attitudes toward Triple P over time, believed the program produces more benefits that outweigh the cost of implementation, and held more optimistic views about program sustainment (Côté & Gagné, 2020b).

Specific characteristics of the evidence-based program itself may also impact its sustainability. For example, program adaptability, program fit within context, research-practice collaboration, as well as the quality of program resources can all influence the programs’ chances of survival (Aarons et al., 2011; Shelton et al., 2018; Turner et al., 2011) found that practitioners were more likely to deliver Triple P if they perceived it as easy to manage and flexible to the diverse needs of clients. Also, practitioners’ ratings on the usefulness of Triple P’s self-regulation framework and whether Triple P’s evidence base is convincing were positively related to their program use (Turner et al., 2011). Lastly, mixed findings suggested contextual factors such as the suitability of the program with clients’ characteristics such as language barriers and complexity of presenting problems can potentially influence program sustainability (Breitkreuz et al., 2011; Sanders et al., 2009).

### The Present Study

Given sustainability is vital for evidence-based programs to achieve community impacts, more studies are needed to address research gaps at the factor-level. One way to extend the literature is to examine predictors of the sustainability of widely implemented evidence-based programs, like the Triple P program, which has international coverage. The overall aim of this study is to investigate predictors of sustained program use, the relative importance of the different factors, and potential mediation paths between factors. All Triple P practitioners who were trained in English-speaking countries in the last 25 years were invited to participate in a large-scale, online international survey about their current program use and factors associated with their delivery. Existing measures of self-efficacy and self-regulation were administered in combination with a newly developed questionnaire designed to comprehensively assess organisational, program-related and other practitioner factors which have been shown to be significant in past research. In addition, archived training data on practitioner self-efficacy was linked to the current survey data and included in analyses.

Using structural equation modelling, a hypothesised model for predicting sustained program use will be created and then tested and modified to identify which model best explains the data. While part of this study is exploratory in nature, based on previous research, we have three hypotheses for the present study:

1. All factors measured in the current survey will predict sustained program use independently, with all being positive predictors except perceived interference.
2. Organisation support will predict most of the other factors including self-regulation; and the effect of self-regulation on sustained program use will be mediated by other surface level factors such as the perceived usefulness of the program.
3. Practitioners’ self-efficacy at early time points will predict later self-efficacy; and as a core component, current self-efficacy will strongly predict current self-regulation.

## Method

### Participants

To investigate sustained use of Triple P, trained practitioners were only included if they had started using the program within 6 months of being trained and had been trained at least one and a half years from the time of data collection. A total of 1202 practitioners who completed their Triple P training between 1997 and 2019 were included in the current study, with 91.3% identifying as female, 7.4% as male, 0.5% as a not listed gender-identity (0.8% did not provide gender information). Most practitioners were aged between 35 and 64 years (83.9%). Practitioners were mostly trained in the United States (34.9%), Canada (23.7%), Australia (19.3%), or the United Kingdom (16.5%). Mental health workers constituted 43.0% of practitioners, 19.6% were teachers/educators, 4.7% were allied health and correction services, 3.7% were medical personnel, and 28.6% did not fit in one of these listed work disciplines. Most practitioners (70.8%) had completed a bachelor’s degree or higher, with 26.7% of the remaining practitioners having completed some tertiary level study. On average, practitioners were trained for six years (*M* = 6.06 years, *SD* = 3.56 years) with about half trained for more than five years, and the majority identified as current users of Triple P (81.4%). Due to the impact of Covid-19, where lockdowns and restrictions interrupted regular service delivery, some practitioners who were categorised as a current user did not use the program in the last six months. About 23.3% of practitioners did not deliver any session in the last six months, 31.5% delivered one to five sessions, 24.6% delivered six to 19 sessions, and 20.5% delivered more than 20 sessions. For detailed information, please see Supplementary Material Table 1.

**Table 1:** Correlations Between Latent Variables

	1	2	3	4	5	6	7	8	9	10	11	12
1. Current Self-Regulation	-											
2. Current Self-Efficacy	0.74^*^	-										
3. Pre-Training SE	0.28^*^	0.34^*^	-									
4. Post-Training SE	0.32^*^	0.36^*^	0.51^*^	-								
5. Follow-Up SE	0.33^*^	0.34^*^	0.36^*^	0.54^*^	-							
6. Organisational Support	0.35^*^	0.23^*^	0.01	0.07	0.08	-						
7. Value Propensity	0.41^*^	0.32^*^	0.08	0.16^*^	0.14^*^	0.30^*^	-					
8. Perceived Usefulness	0.58^*^	0.39^*^	0.05	0.11^*^	0.16^*^	0.51^*^	0.45^*^	-				
9. Perceived Interference	− 0.13^*^	− 0.13^*^	− 0.05	− 0.11^*^	− 0.09	− 0.16^*^	− 0.13^*^	− 0.12^*^	-			
10. Satisfaction with Program Features	0.48^*^	0.36^*^	0.10	0.18^*^	0.20^*^	0.45^*^	0.44^*^	0.62^*^	− 0.19^*^	-		
11. Session Management Ability	0.49^*^	0.37^*^	0.10	0.12^*^	0.16^*^	0.43^*^	0.29^*^	0.48^*^	− 0.12^*^	0.47^*^	-	
12. Frequency of Use	0.30^*^	0.22^*^	0.07	0.11^*^	0.09	0.39^*^	0.11^*^	0.35^*^	− 0.13^*^	0.19^*^	0.27^*^	-
95% CI (Lower)	0.25	0.16	0.02	0.05	0.03	0.34	0.05	0.30	− 0.18	0.14	0.22	-
95% CI (Upper)	0.35	0.27	0.13	0.16	0.15	0.44	0.17	0.40	− 0.07	0.25	0.32	-
*M*	5.75	5.85	4.49	5.79	6.15	5.02	6.57	5.52	2.15	5.81	5.11	2.60
*SD*	0.92	1.09	1.18	0.78	0.67	1.58	0.70	1.19	1.21	1.13	1.44	2.28

*Note.* Variable 1 to 11 range from 1 to 7. Variable 12 range from 0 to 7. All correlation coefficients are Spearman’s *rho*. The 95% confidence interval is for correlations with Frequency of Use, with Fisher’s method

^*^p < .001

### Procedure

All Triple P practitioners who were trained in English-speaking countries were sent an email invitation to complete a 10-minute online survey. Emails were sent out by Triple P International, the training organisation who has permission to contact practitioners, in May 2021. Two reminder emails were sent, one per week in the two weeks following the initial email. Emails were successfully delivered to 28,789 practitioners, where 13,371 opened the email, and 2,663 clicked the survey link. Archived data was also included in the study from measures of practitioner self-efficacy completed by practitioners during professional training, which was provided by Triple P International and contained identification numbers which were then used to link this data with the responses to the current survey.

### Measures

The current questionnaire contained four sections: a demographic questionnaire, a brief program use survey, measures of practitioners’ self-efficacy and self-regulation, and the facilitators and barriers checklist. The same self-efficacy measure was used to assess practitioner’s self-efficacy before, immediately after and one month after the training.

#### Sustained Program Use

Two aspects of sustained program use were measured, namely user status and frequency of use. User status was defined as a practitioner who had started using the program within the first 6 months post-training and who still considered themselves to be using the program currently. Practitioners who said yes to using Triple P within the first six months after training, were asked whether they had stopped using Triple P or not. User status was coded as a dichotomous variable where a practitioner who had not stopped using was coded as a current user (coded as 1) and a practitioner who had stopped using was coded as a stopped user (coded as 0). Frequency of use was measured with one question, “*Making an estimation, about how many sessions of Triple P did you deliver in the last six months?*”, which required rating on an 8-point scale from 0 to 7 (see *Appendix* for response options).

#### Self-Efficacy

Practitioners’ current self-efficacy, as well as their self-efficacy pre-, post- and one-month-following training were assessed with the Practitioner Confidence subscale of the Parent Consultation Skills Checklist (PCSC; Turner & Sanders 1996). This subscale contains two items, each rated on a Likert scale ranging from 1 (not at all proficient) to 7 (extremely proficient, no assistance required). The items were “*How confident are you in conducting parent consultations about child behaviour?*” and “*Do you feel adequately trained to conduct parent consultations about child behaviour?*”. Responses to the two items were averaged to create a scale score, with higher score indicating higher self-efficacy. In the current study, the scale displayed good to excellent internal consistency (pre-training: α = 0.89; post-training: α = 0.85; follow-up: α = 0.82; current: α = 0.94).

#### Self-Regulation

Practitioners’ current self-regulation was measured by the practitioner version of the Parenting Self-Regulation Scale (PSRS-Practitioner; Sanders et al., 2017). PSRS-Practitioner contains 12 items rated on a Likert scale from 1 (strongly disagree) to 7 (strongly agree). The scale has a single-factor structure (Tellegen et al., 2021), and demonstrated excellent internal consistency in the current study (α = 0.96). Sample items were “Q6: *I have the skills to be an effective Triple P provider*” and “Q11: *I can apply what I learn about parenting interventions to different situations*”. All items were positively worded, and a total score was calculated by averaging all 12 items. Higher scores reflect higher self-regulation in delivering Triple P.

#### Facilitators and Barriers of Sustained Program Use

For this study, we developed a 51-item questionnaire called the *Facilitators and Barriers Checklist* (FBC) to assess a wide range of factors that may potentially impact sustained program use. Items were mainly taken from previous Triple P implementation studies (e.g. Hodge et al., 2017; Sanders et al., 2009; Shapiro et al., 2012), and six items measuring practitioners value propensity were adapted from core values underpinning the Triple P approach (Sanders, 2018). Through logistic regressions and factor analyses, we reduced the number of items down to 39. Items were rated was from 1 (strongly disagree) to 7 (strongly agree). The validation process, full items for each subscale, and the whole item pool are presented in *Supplementary Material*. The *FBC* has six subscales, namely organisational support (14 items; e.g. “Q19: *Delivering Triple P is emphasised and encouraged at my organisation*”), value propensity (6 items; e.g. “Q10: *I strongly believe that raising healthy well-adjusted children is a shared responsibility among all the carers in a child’s life*”), perceived usefulness (6 items; e.g. “Q5: *I think Triple P is producing observable change in children and families*”), perceived interference (6 items; e.g. “Q31: *Offering Triple P interferes with my personal free time*”), satisfaction with program features (4 items; e.g. “Q38: *Research evidence regarding program effectiveness is convincing to me*”), and session management ability (3 items; e.g. “Q45: *I can keep parents on track during consultations*”). The internal consistency of each subscale ranged from acceptable to excellent (α = 0.95, 0.92, 0.83, 0.77, 0.84, and 0.78 respectively). All subscales were positively coded except for program interference and subscale totals were an average score of the items.

### Analysis

The factor structure for the *Facilitators and Barriers Checklist* was tested first (see *Supplementary Material*) to determine the number of latent factors to include in further Structural Equation Modelling (SEM) analysis to test a model of the relationships among study variables. In the preliminary analyses, we first examined the correlation between latent factors and frequency of use, and logistic regressions of each latent factor on user status. The hypothesised model of the latent factors was created based on theoretical considerations. When research gaps remain around certain factor, we occasionally consulted the correlation matrix between latent factors. A regression path was only established if it is theoretically justifiable and the correlation between two factors exceeded medium level (0.30 according to Cohen 1988). To evaluate the model fit, the chi-square (χ^2^), the comparative fit index (CFI), the root-mean-squared error of approximation (RMSEA), and the Standardized Root Mean Squared Residual (SRMR) were used following guidelines for suggested cut-off values (Blunch, 2008; Byrne, 2010). The ideal cut-off values are *p* > .05 for χ^2^, CFI > 0.95, RMSEA < 0.05, and SRMR < 0.08. Moreover, models showing CFI > 0.90, and RMSEA < 0.08 are acceptable (Blunch, 2008; Byrne, 2010). Also, it is typical in social science research to find a statistically significant χ^2^ due to its sensitivity to large samples (Byrne, 2010). If the initial model fitted inadequately, the standardised residuals and the modification indexes were calculated to detect model misspecification, following the procedure proposed by Byrne (2010). SEM analyses were conducted with the *Lavaan* package in *R Studio*. To accommodate the large number of analyses, we set the significance level at *p* < .001.

## Results

### Missing Data Analysis

This study is part of a larger implementation project with data from 1618 participants. To specifically examine sustained use in the current study, only participants who were trained at least one and a half years before data collection and who had initiated program use were included in the dataset, leaving a total of 1202 participants. Approximately 65% of participants had no data missing, less than 3% had more than 10% data missing, and less than 1% had more than 20% data missing. We deleted all 12 participants with more than 20% data missing. No variable had more than 20% of missing data. A significant Little’s MCAR test, χ2(16,903, *N* = 1606) = 17815.75, *p* < .001, suggested that the data was not missing completely at random. Separate variance t-tests suggested that the missingness of some variables was influenced by other variables within the study, indicating the data was missing at random (MAR; Bennett 2001). Therefore, we used expectation-maximisation algorithm to substitute all missing values for continuous variables (Bennett, 2001).

### Preliminary Analysis

Correlations between study variables are presented in Table 1. The majority of correlations were significant and ranged from small to large (Cohen, 1988). Frequency of use was correlated with most of the variables except self-efficacy at pre-training and at follow up. A series of independent logistic regressions was conducted to examine the relationship between different factors and user status. Eight factors were significant predictors, where seven were positive (current self-regulation, current self-efficacy, organisational support, value propensity, perceived usefulness, satisfaction with program features, and session management ability) and one was negative (perceived interference). Self-efficacy before training, immediately after training and at one-month follow-up were not significant predictors. All statistics and coefficients are displayed in Table 2.

**Table 2:** Logistic Regressions of Predictors of User Status

Predictor	Model fit (χ2)	Cox & Snell	Nagel-kerke	*Wald*	*Exp*(*B*)	95%CI
Current Self-Regulation	57.10	0.05	0.08	55.38	1.788^*^	1.534–2.084
Current Self-Efficacy	14.80	0.01	0.02	15.24	1.285^*^	1.133–1.457
Pre-Training Self-Efficacy	0.31	0.00	0.00	0.31	1.036	0.916–1.171
Post-Training Self-Efficacy	0.11	0.00	0.00	0.11	1.032	0.858–1.241
Follow Up Self-Efficacy	0.04	0.00	0.00	0.04	0.979	0.789–1.216
Organisational Support	233.11	0.18	0.29	188.57	2.069^*^	1.865–2.295
Value Propensity	19.97	0.02	0.03	20.55	1.527^*^	1.272–1.833
Perceived Usefulness	188.77	0.15	0.24	150.65	2.342^*^	2.044–2.683
Perceived Interference	23.51	0.02	0.03	24.42	0.758^*^	0.679–0.846
Satisfaction with Program Features	57.82	0.05	0.08	56.71	1.591^*^	1.410–1.795
Session Management Ability	136.13	0.11	0.17	121.70	1.786^*^	1.611–1.980

^*^p < .001

### Constructing the Hypothesised Model

The hypothesised model was created after determining the factor structure of the *FBC* because it was newly developed for this study. The model was created based on theoretical considerations and investigating the correlation table. Given previous waves of self-efficacy were not associated with current program use and were only predicting current self-efficacy, they were excluded from the model. Given self-efficacy is considered to be a major component of self-regulation and the high correlation between the two variables, the current self-regulation latent factor in the model is combined with items from both current self-efficacy measure and self-regulation measure. Organisational support is external to the practitioner, so it was therefore hypothesised to be a predictor of (and not predicted by) the other practitioner-related variables. Organisational support was expected to predict both current self-regulation and satisfaction with program features because practitioners are more likely to develop skills and be convinced by the program quality in supportive organisations. The direction of the prediction relationship between self-regulation and satisfaction with program features remained unclear. The remaining three factors, namely perceived usefulness, session management ability and value propensity were expected to be predicted by organisational support, self-regulation, and satisfaction with program features but not predicting other factors. This is because these three factors are independent surface-level factors that are influenced by higher level factors such as their self-regulation capacity in delivery. Perceived interference was not predicted by other factors. The direct paths between all factors and two sustained program use variables were examined. A hypothesised regression path would only be formed if the correlation between two factors exceeds 0.30. The hypothesised model is displayed in *Supplementary Material*.

### The Measurement Model

The measurement model was evaluated first by allowing all latent factors to correlate with each other. We used MLR estimator in the *Lavaan* package in *R Studio.* The initial model did not fit well. After investigating the modification indices and checking the validation processes for the *PSRS – Practitioner* and the *FBC*, three covariations between similar items were added, one at a time (for detail, see *Supplementary Material*). The modified model (Model C) fit the data adequately well, χ^2^(1301) = 6249.41, *p* < .001, CFI = 0.901, SRMR = 0.054, RMSEA = 0.054 [90%CI: 0.053-0.056]. All factor loadings were significant at *p* < .001 level and range from 0.35 to 0.91.

### The Structural Model

**Fig. 1:**
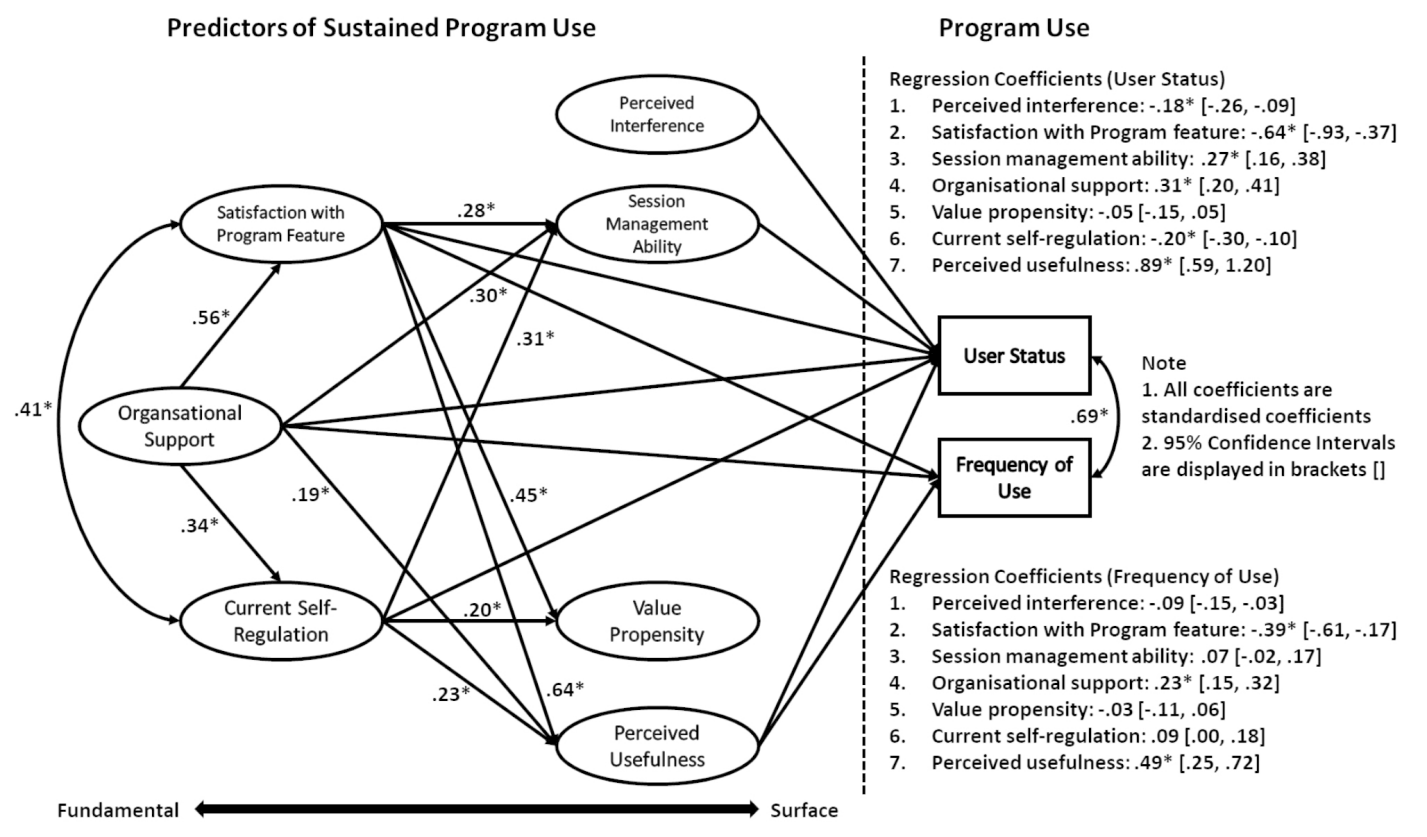
Final Model of Different Factors on the Sustained Use of the Triple P Program *Note*: Only significant predictions are displayed in the figure. Circles represent latent variables that contain individual items. ^*^p < .001.

Given one of the dependent variables (user status) was a dichotomous categorical variable, we used weighted least square mean and variance adjusted estimator (WLSMV) in the *Lavaan* package in *R Studio*, which is the most appropriate estimation method for dichotomous variables (Brown, 2006). All predictor variables were examined as latent factors, while program use variables were examined as observed variables. The hypothesised model was tested and did not fit sufficiently well, χ^2^(1406) = 4632.31, *p* < .001, CFI = 0.871, SRMR = 0.054, RMSEA = 0.036 [90%CI: 0.035-0.037]. Given we used a different estimation method (WLSMV) to the measurement model evaluation and the validation process of other measures included, as demonstrated in Table 3, we made ten modifications (deleted three items and added seven correlations between items), one at a time, based on modification indexes and theoretical considerations. The final model of sustained program use fitted well, χ^2^(1243) = 2804.67, *p* < .001, CFI = 0.900, SRMR = 0.046, RMSEA = 0.033 [90%CI: 0.031-0.034]. The final model and all coefficients are displayed in Fig. 1. It explained 55% of the variance in user status and 24% of the variance in frequency of use. Note that, because no statistical software could provide logistic regression output for a binary dependent variable (user status) in SEM, the regression coefficients reported in the model are probability regression coefficients.

**Table 3:** Fit Indices for the Structural Model

Model Number	χ^2^	*df*	CFI	SRMR	RMSEA	RMSEA 90% CI
Original Model	4632.31^*^	1406	0.871	0.054	0.036	0.035-0.037
A: Deleted PSRS 8	4168.55^*^	1353	0.880	0.052	0.035	0.033-0.036
B: Deleted FBC 43	3702.78^*^	1301	0.883	0.051	0.035	0.033-0.036
C: Added correlation between PCSC 1 with PCSC 2	3605.34^*^	1300	0.887	0.050	0.034	0.033-0.036
D: Deleted FBC 40	3069.68^*^	1249	0.889	0.047	0.034	0.033-0.036
E: Added correlation between FBC 11 with FBC 12	3020.22^*^	1248	0.891	0.047	0.034	0.032-0.036
F: Added correlation between FBC 10 with FBC 12	2958.27^*^	1247	0.894	0.047	0.034	0.032-0.035
G: Added correlation between FBC 11 with FBC 12	2881.92^*^	1246	0.897	0.047	0.033	0.031-0.035
H: Added correlation between FBC 2 with FBC 3	2847.10^*^	1245	0.898	0.046	0.033	0.031-0.034
I: Added correlation between PSRS 2 with PSRS3	2824.55^*^	1244	0.899	0.046	0.033	0.031-0.034
J: Added correlation between PSRS 11 with PSRS 12	2804.67^*^	1243	0.900	0.046	0.033	0.031-0.034

*Note.* PSRS: Parenting Self-Regulation Scale – Practitioner Version, FBC: Facilitators and Barriers Checklist, PCSC: Practitioner Consultation Skill Checklist

^*^p < .001

In the final model, two factors were unique positive predictors of both sustained program use variables, namely perceived usefulness of the program and organisational support. Session management ability was a positive predictor of user status but not the frequency of use. However, the direction of the effect of satisfaction with program features changed to negative on both outcome variables after controlling for the other factors and mediation paths on both outcome variables, while current self-regulation changed to negative for user status and non-significant for frequency of use. Perceived interference negatively predicted both outcomes. Value propensity did not significantly predict any outcome in the final model. Moreover, organisational support positively predicted self-regulation.

The mediation indirect effects were calculated for important pathways. Perceived usefulness partially mediated the effect of satisfaction with program features (on user status: *β* = 0.57, *SE* = 0.15, *p* < .001, 95% CI [0.36, 0.79]; on frequency of use: *β* = 0.31, *SE* = 0.08, *p* < .001, 95% CI [0.15, 0.47]), organisational support (on user status: *β* = 0.17, *SE* = 0.04, *p* < .001, 95% CI [0.10, 0.24]; on frequency of use: *β* = 0.09, *SE* = 0.03, *p* < .001, 95% CI [0.04, 0.14]), and current self-regulation on sustained program use (on user status: *β* = 0.21, *SE* = 0.04, *p* < .001, 95% CI [0.14, 0.28]; on frequency of use: *β* = 0.11, *SE* = 0.03, *p* < .001, 95% CI [0.06, 0.17]). Session management ability mediated the effects of satisfaction with program features (*β* = 0.08, *SE* = 0.02, *p* < .001, 95% CI [0.04, 0.11]), organisational support (*β* = 0.08, *SE* = 0.02, *p* < .001, 95% CI [0.05, 0.12]), and current self-regulation on user status (*β* = 0.08, *SE* = 0.02, *p* < .001, 95% CI [0.05, 0.12]). The relationship between organisational support and perceived usefulness of the program is mediated by satisfaction with program features (*β* = 0.36, *SE* = 0.03, *p* < .001, 95% CI [0.31, 0.41]), and current self-regulation (*β* = 0.08, *SE* = 0.01, *p* < .001, 95% CI [0.06, 0.10]).

## Discussion

This exploratory study aimed to identify predictors of sustained program use of an evidence-based parenting program, Triple P, as well as to examine the relative importance of different factors and the mediation relationships between factors. We utilized large-scale, international survey data to create and estimate a structural equation model for predicting practitioners’ sustained program use. About half of the sample were accredited to deliver the program for more than five years. The current model explained considerable proportions of variance in both sustained program use outcomes, namely user status and frequency of use. The present model accounted for more variance in sustained program use than a previous structural equation model of Triple P implementation (55% in user status and 24% in frequency of use versus 9%; Turner et al., 2011).

The first hypothesis was supported where all current factors predicted sustained program use independently, with all being positive except perceived interference. Organisational support was one of the strongest independent predictors of practitioners’ frequency of delivery and whether the practitioner considered themselves as a current user. Consistent with previous literature (Asgary-Eden & Lee, 2012; Hodge et al., 2017), practitioners were more likely to use the program over time when they perceived that their organisations recognise, support, and encourage Triple P delivery. As expected, practitioners’ perceived usefulness of Triple P was a top independent positive predictor of sustained program use. In line with previous findings, where practitioners reported that the most important reason for sustained Triple P delivery was simply “it works” (Shapiro et al., 2015, p. 1621). Highly self-regulated and self-efficacious practitioners were more likely to continue delivering the program, which is consistent with previous studies where self-efficacy could predict post-training program use (Sanders et al., 2009; Turner et al., 2011), and was recognized by practitioners as a major contributor to sustained delivery (Shapiro et al., 2015).

Additionally, practitioners’ self-reported session management ability was a facilitator of sustained program use. The positive prediction was anticipated as this concept is closely related to self-efficacy. As expected, and suggested by sustainability frameworks (Aarons et al., 2011; Shelton et al., 2018), practitioners’ satisfaction with program features such as the resources quality, adaptability, and evidence base predicted sustained program use. Also, sustained program use was positively linked with practitioners’ propensity of agreeing with the six core values of the Triple P approach (Sanders, 2018), which provided support to the inclusion of personal values and attitudes in many implementation frameworks (Damschroder et al., 2009; Shelton et al., 2018). Lastly, congruent with literature (Sanders et al., 2009; Shapiro et al., 2015), practitioners tended to deliver the program less if they thought the delivery interfered with their work schedule, personal life, or preferred theoretical approach.

In the structural model, as expected in hypothesis two, organisational support fostered sustained Triple P use directly, and positively predicting other potential facilitators such as practitioners’ self-regulatory capacity in program delivery, satisfaction with the program features, session management ability, and personal beliefs about the program usefulness. The prediction path of self-regulation confirmed the findings of a previous study, where self-efficacy mediated the effect of practitioner self-reported organisational implementation climate (e.g. prioritising program use) on sustained program use (McWilliam, 2016). In addition, the prediction of other potential facilitators could explain the findings of another study, where practitioners perceived training needs (which could relate to self-regulation, session management ability, and perceived program usefulness) decreased along with more program delivery, especially when the perceived organisational capacity was high (Côté & Gagné, 2020a).

Organisational support was found to be a key facilitator of program sustainability in both preliminary analyses and the structural equation model. Besides its positive impact on other factors, the significant direct path indicated that organisational support had independent positive effects on sustained program use that were over and above other practitioner and program-related factors. This emphasises the need for service organisations and program purveyors (as well as program developers and researchers) to develop collaborative partnerships with program purveyors to maintain their capacity at an optimal level (Hodge & Turner, 2016). This is consistent with how Triple P is disseminated, with the purveyor organisation using the Triple P Implementation Framework to provide support to organisations across different phases of implementation, from early engagement to post implementation maintenance (McWilliam et al., 2016).

Another key facilitator was practitioners’ perceived usefulness of Triple P, which was the strongest predictor in the model. Practitioners tend to personally evaluate the benefit-to-cost ratio of the program (Hodge & Turner, 2016); thus noticeably effective and appealing programs are more likely to survive over time (Shapiro et al., 2015). Interference caused by program use might be analysed as a burden in practitioners’ own cost efficiency evaluation of the program, which in turn became an inhibitor of continued delivery (Sanders et al., 2009; Shapiro et al., 2015). In the model, perceived usefulness was predicted by both current self-regulation and satisfaction with program features including satisfaction with the evidence base. This suggested that, as described in the scientist-practitioner model (Jones & Mehr, 2007), practitioners might evaluate the program based on both research-based evidence such as clinical trials, and practice-based evidence gathered in their own delivery. It is desirable for evidence-based public health programs such as parenting programs to have excellent benefit-to-cost ratio to both service organisations and practitioners to increase sustainability.

Additionally, in support of hypothesis two, self-regulation positively predicted facilitators of sustained use, such as session management ability and perceived program usefulness. Highly self-regulated practitioners tended to produce more successful client outcomes and receive more positive feedback (Sanders & Turner, 2005), which in turn meant they became more likely to use the program over time (Turner et al., 2011). Surprisingly, the direct effect of self-regulation on practitioners’ self-classification current user status became negative, which might reflect that some highly self-regulated practitioners tended to apply their Triple P consultation skills more flexibly and less programmatical, thus did not consider themselves as current users.

The third hypothesis about practitioners’ self-efficacy and self-regulation in program delivery was supported. Consistent with previous literature (Shapiro et al., 2012), self-efficacy at post-training follow-up was positively linked to pre-training and immediate post-training self-efficacy, and was positively associated with current self-efficacy, a facilitator of sustained program use. This suggested high-quality professional training might enhance program sustainability by making practitioners feel more confidence in delivering the program. However, all three self-efficacy ratings collected during practitioner training were unrelated to or only weakly related to sustained program use independently. This is contrary to a previous study where practitioners post-training self-efficacy were related to their program use two years later (Shapiro et al., 2012). This might suggest that the proximal environment is more important to the survival of the implementation than the influence of historical factors such as the quality of training. The importance of post-implementation support is further stressed (McWilliam et al., 2016). Also, in support of the self-regulation framework where self-efficacy is one of the five core components (Sanders & Mazzucchelli, 2013; Sanders et al., 2019), current self-efficacy was strongly positively associated with practitioners’ current self-regulation. Furthermore, the self-regulation measure (i.e. PSRS; Sanders et al., 2017) generally showed a stronger association with most of the other factors including sustained program use than the self-efficacy measure (i.e. PCSC; Turner & Sanders 1996) that is currently used in practitioner training (Sethi et al., 2014; Shapiro et al., 2008). Given self-regulation is a fundamental principle underlying Triple P, the PSRS should be considered as potential measure to evaluate training outcomes.

The present study included a large, multidisciplinary, international sample of Triple P practitioners who received their initial training between 1997 and 2019 in major English-speaking countries. Almost half of the sample were trained more than five years prior to data collection (May, 2021). Given most previous research used single-site studies with relatively brief measurement periods (Shelton & Lee, 2019), the inclusion of practitioners with variable length of implementation periods provided a more accurate representation of the dynamic nature of sustainability where the importance of different factors may change over time (Stirman et al., 2012). The current design balanced robustness and feasibility.

Several limitations need to be mentioned. One primary limitation is the dependence on practitioners’ self-report data on all variables including the retrospective estimation of program use. Although using self-report measures is more feasible and practical for large-scale international studies, it is vulnerable to response bias, and has limits in presenting objective information about program use and organisational capacity. Future studies should include more objective measures of program use (e.g. case files, rebate records; for example, see Brookman-Frazee et al., 2018; Lau et al., 2021), additional independent assessment of organisational capacity, as well as fidelity (e.g. observation). Second, although there was archival data on practitioner self-efficacy included in the model, there was not archival data available on the other factors of interest. Therefore, most of the data analysed was based on a single cross-sectional survey, therefore limiting drawing definitive conclusions about the causal nature of the relationships discussed and mediation pathways evaluated (Maxwell & Cole, 2007; Maxwell et al., 2011). Although the use of cross-sectional data has advantages in feasibility and practicality, future research using longitudinal data would help to draw more definitive conclusions about the nature of the relationships between the predictors studied in this model. Third, generalisation of the findings needs to be dealt with cautiously as the sample may not be representative of all practitioners. All survey respondents were from English-speaking developed countries, with over 90% identifying as female and over 70% with university degrees. The proportions of practitioners filled into each gender and education category were consistent between the current study and the proportions reported in a larger study using the entire archived dataset of practitioners trained in Triple P (Sanders et al., 2022). Although these percentages are typical for this practitioner group, the pattern of findings may not be generalisable to the entire population of practitioners. Although emails were successfully delivered to 28,789 practitioners only 46% opened the email and of these, data from only 9% of this sample was analysed in this study. The practitioners who completed the survey may not be representative of the population and are perhaps more likely to hold polarised views about Triple P and its delivery, given no incentive was provided for participation. Fourth, the current model did not contain any contextual predictor of sustainability. Although several client-related items such as experiencing unavailability of clients were barriers of sustained program use, and several program fit with the context items such as program’s appropriateness for clients presenting problems were facilitators of program use, they did not load on any of the factors in the structural equation model and were thus excluded (see Supplementary Material). Future work should include more items to capture the contextual influence such as socio-political environment, funding sources, community characteristics, and the fitness of the program with the context. Lastly, practitioners’ frequency of program use might be impacted by the Covid-19 pandemic where lockdowns and restrictions were in place and be less reflective of normal practice. Also, the diverse Triple P modes of delivery (including individual, group, and large group seminar sessions; Sanders 2012) increased the heterogeneity in the frequency of use variable. To address the impact of the pandemic and different delivery modes, we added self-report user status in addition to frequency of use to measure sustainability. The results on both sustained program use indicators were largely consistent, strengthening the conclusions which could be drawn.

This exploratory study sought to identify factors influencing the sustained program use of Triple P, and to investigate the relative importance of different factors and the relationships between factors. Three key findings were obtained. First, organisational support was found to be central to sustainability. It was not only positively associated with a range of other facilitators such as self-regulation and perceived usefulness but had independent facilitating effects on sustained program use above other factors. Second, practitioners’ perceived usefulness of the program was the most important practitioner level facilitator of sustained program use. Practitioners were more likely to use the program when they were convinced by both research-based and practice-based evidence. Third, self-regulation impacted sustained program use through influencing other factors such as perceived usefulness of the program. The self-regulation measure used in the current study could be considered as an alternative to evaluate training outcomes.

## Electronic Supplementary Material

Below is the link to the electronic supplementary material.

Supplementary Material 1
