# Supplementary material for: Predicting the Sustained Implementation of an Evidence-Based Parenting Program: A Structural Equation Modelling Approach
Source: Adm Policy Ment Health. 2022 Nov 5;50(1):114–27. doi: 10.1007/s10488-022-01226-x (PMC9638368; doi:10.1007/s10488-022-01226-x)
Supplement: Supplementary file 1 — Supplementary Material 1 [file 10488_2022_1226_MOESM1_ESM.docx]

**Supplementary Material**

**Detailed Demographic**

**Table 1:**

*Sample Demographics*

| Category | Frequency | Percentage (%) |
| --- | --- | --- |
| **Age** | | |
| 20-24 | 2 | 0.2 |
| 25-29 | 42 | 3.5 |
| 30-34 | 74 | 6.2 |
| 35-39 | 135 | 11.3 |
| 40-44 | 164 | 13.7 |
| 45-49 | 180 | 15.0 |
| 50-54 | 208 | 17.3 |
| 55-59 | 185 | 15.4 |
| 60-64 | 136 | 11.3 |
| 65-69 | 52 | 4.3 |
| 70 and above | 21 | 1.7 |
| Missing | 3 | 0.2 |
| **Gender** | | |
| Male | 89 | 7.4 |
| Female | 1097 | 91.3 |
| A not listed gender-identity | 6 | 0.5 |
| Missing | 10 | 0.8 |
| **Education** | | |
| High School | 24 | 2.0 |
| Some College/University study | 321 | 26.7 |
| Bachelor’s degree | 480 | 39.9 |
| Master’s degree | 335 | 27.9 |
| Doctorate/PhD/MD | 36 | 3.0 |
| Missing | 6 | 0.5 |
| **Discipline** | | |
| Allied Health and Correction Services | 57 | 4.8 |
| Teachers/Educators | 236 | 19.7 |
| Mental Health Workers (Including Psychologists, Social Workers and Counsellors) | 517 | 43.2 |
| Medical Personnel | 45 | 3.8 |
| Others | 341 | 28.5 |
| Missing | 6 | 0.5 |
| **Year of Initial Training** | | |
| Prior 2012 (1997-2011) | 168 | 14.0 |
| 2012 | 117 | 9.7 |
| 2013 | 98 | 8.2 |
| 2014 | 123 | 10.2 |
| 2015 | 91 | 7.6 |
| 2016 | 131 | 10.9 |
| 2017 | 157 | 13.1 |
| 2018 | 150 | 12.5 |
| 2019 | 167 | 13.9 |
| **Training Region** | | |
| Australia | 232 | 19.3 |
| Canada | 285 | 23.7 |
| Ireland | 6 | 0.5 |
| New Zealand | 52 | 4.3 |
| United Kingdom | 198 | 16.5 |
| United States | 420 | 34.9 |
| Others (Denmark, Costa Rica) | 9 | 0.8 |
| **User Status** | | |
| Stopped User | 224 | 18.6 |
| Current User | 978 | 81.4 |
| **Frequency of Use in the Last Six Months** | | |
| 0 Session | 280 | 23.3 |
| 1-2 Sessions | 202 | 16.8 |
| 3-5 Sessions | 177 | 14.7 |
| 6-9 Sessions | 150 | 12.5 |
| 10-19 Sessions | 146 | 12.1 |
| 20-29 Sessions | 79 | 6.6 |
| 30-39 Sessions | 33 | 2.7 |
| More Than 40 Sessions | 135 | 11.2 |

*Note.* Due to rounding, the percentage may not sum up to 100. Due to the impact of Covid-19, where lockdowns and restrictions interrupted regular service delivery, some practitioners who were categorised as a current user did not use the program in the last six months.

**Validation of the Facilitators and Barriers Checklist**

Because the *Facilitators and Barriers Checklist* was developed for the purpose of this study, we first needed to understand its factor structure before using it for SEM. Given the questionnaire was developed without a theoretical model, we first conducted EFA, followed by CFA. Independent binary logistic regressions revealed that five items did not significantly predict user status at *p* < .05 level (Q20, Q29, Q30, Q46, and Q49), with the lowest *p*-value for Q46, *B* = 0.05, *SE* = 0.05, *Wald* = 1.13, *p* = .287. Thus, we excluded these five items from the factor analyses. The remaining 46 items were tested for skewness and kurtosis. As we expected, most positively worded items were negatively skewed, and negatively worded items were positively skewed. The average absolute skewness was 1.32, and six out of the 46 items showed significant skewness with absolute values above 2.1 (West et al., 1995). All six items captured practitioners value propensity (i.e., question 7-12). The average absolute kurtosis was 2.16 and five items of practitioners’ value showed significant kurtosis values above 7 (West et al., 1995).

Next step, we examined the factorability of the items. The correlation matrix displayed adequate correlations between items, the value of Kaiser-Meyer-Oklin measure of sample adequacy was .94 (greater than the conventional .90 threshold), and the results of Bartlett’s test of sphericity was significant, χ2 (1035, N = 1202) = 32265.75, p < .001. These all indicated suitability for factor analysis (Field, 2018). Then, we randomly split our sample into two (EFA = 603, CFA = 599). This satisfied the range of suggested minimum sample sizes including minimum 300 participants (MacCallum et al., 1999), 5:1 participant to item ratio (Gorsuch, 1983), and 10:1 participant to item ratio (Conrad & Mario, 2014).

***Exploratory Factor Analysis***

We first used principal component analysis (PCA) to determine the number of factors to extract. Scree plots suggested a six-factor structure (see *Figure 1*), while Kaiser’s criterion of eigenvalues greater than one, suggested a nine-factor structure. After investigation, we decided to proceed with the six-factor structure to have the most parsimonious model. To address skewness and kurtosis, we used the principal axis factoring extraction method with specifying the number of factors to extract, which is preferred for non-normality of observations (Fabrigar et al., 1999). Additionally, an oblique (Promax) rotation method was chosen to enhance the interpretability of our factor structure because factors are likely to be correlated with each other.

Items with loadings of less than .40 on any factor were excluded one by one from the EFA. We excluded seven items, namely Q51, Q50, Q35, Q41, Q4, Q13, and Q48, all listed in the step order. The final model consisted of 39 items. The six-factor structure accounted for 57% of the total variance. The factor loading of each item, the eigenvalue of each factor, and the percentage of variance accounted for by each factor are displayed in *Table 2*. All items had at least .40 loading on a factor. All items included in the final model had adequate communalities (range from .27 to .84) and extraction rate (range from .26 to .81). One item which had cross loading between .30 and .40 on other factors, was retained. All factors were correlated with each other, with the correlation coefficient ranging from .09 to .59.

**Table 2**

*Exploratory Factor Analysis for the Facilitators and Barriers Checklist*

| Item Number and Name | Factor loading | |
| --- | --- | --- |
| Factor one: Organisational support | |  |
| 15. Staff in my organisation are supportive of offering the Triple P program for parents in the community | .75 | |
| 16. My organisation commits resources to planning and providing the Triple P program | .87 | |
| 17. My organisation is supportive of professional development around Triple P | .88 | |
| 18. My organisation has an adequate physical environment for offering Triple P | .66 | |
| 19. Delivering Triple P is emphasised and encouraged at my organisation | .87 | |
| 21. Supervision or case discussions have been helpful for the use of Triple P | .63 | |
| 22. My supervisor is supportive of offering the program for parents in the community | .86 | |
| 23. My work with the program is viewed positively by my supervisor | .83 | |
| 24. My supervisor has brought in expertise to support my delivery of Triple P | .73 | |
| 25. My Triple P work is recognised and valued by colleagues | .67 | |
| 26. I receive support from others in my community for my work | .61 | |
| 27. I consult with other Triple P practitioners | .64 | |
| 28. While working on the program, I am able to rely on my co-workers for ideas and support | .77 | |
| 36. I have access to support when I have questions or need support for my Triple P delivery | .53 | |
| Eigenvalue | 13.03 | |
| Total variance accounted (%) | 33.40 | |
| Factor two: Value propensity | |  |
| 7. I strongly believe all parents should have the opportunity to participate in a high-quality, evidence-based parenting program to support their children’s healthy development and well-being. | .71 | |
| 8. I strongly believe that parenting programs should enable equality of access for both genders and all family types, cultures, religions, and political beliefs. | .80 | |
| 9. I am deeply convinced that positive parenting programs should adopt an empowerment approach that is built strongly on a self-regulation framework. This self-regulation framework respects a parent’s capacity to learn the necessary skills needed, and respects a parent’s right to make informed decisions about how to raise his or her children. | .73 | |
| 10. I strongly believe that raising healthy well-adjusted children is a shared responsibility among all the carers in a child’s life. | .89 | |
| 11. I strongly believe positive parenting programs should seek to create child-, parent-, and family-friendly communities. | .87 | |
| 12. I firmly believe that children are our communities’ most valued resource, and we need to ensure their well-being by building a nurturing, caring environment. | .80 | |
| Eigenvalue | 3.86 | |
| Total variance accounted (%) | 8.97 | |
| Factor three: Perceived usefulness | | |
| 1. I give out tip sheets or show videos to parents | .45 | |
| 2. I provide Triple P advice to neighbours, friends, adult family members, or anyone else in something other than your normal work setting | .56 | |
| 3. I incorporate Triple P ideas or principles into my work in general | .70 | |
| 5. I think Triple P is producing observable change in children and families | .74 | |
| 6. I receive positive feedback from parents regarding the program | .70 | |
| 14. I identify myself as a Triple P provider | .61 | |
| Eigenvalue | 2.91 | |
| Total variance accounted (%) | 6.11 | |
| Factor four: Perceived interference | | |
| 31. Offering Triple P interferes with my personal free time^#^ | .74 | |
| 32. After hours appointments clash with my other commitments^#^ | .76 | |
| 33. I experience unavailability of overtime or time off in lieu (compensatory time) for after-hours appointments^#^ | .57 | |
| 40. Triple P delivery interferes with my work schedule and responsibilities^#^ | .67 | |
| 42. Triple P does not integrate with my caseload or other responsibilities at work^#^ | .50 | |
| 43. The theoretical approach of Triple P clashes with my theoretical perspective or preferred treatment approach^#^ | .42 | |
| Eigenvalue | 2.15 | |
| Total variance accounted (%) | 4.50 | |
| Factor five: Satisfaction with program features | | |
| 34. I think Triple P parent and practitioner materials are helpful | .53 | |
| 37. Research evidence regarding program effectiveness is convincing to me | .69 | |
| 38. I think Triple P’s self-regulatory framework is useful | .76 | |
| 39. I think Triple P is easy to tailor to the needs of individual families | .56 | |
| Eigenvalue | 1.41 | |
| Total variance accounted (%) | 2.58 | |
| Factor six: Session management ability | | |
| 44. I am able to cover session material in the scheduled time | .72 | |
| 45. I can keep parents on track during consultations | .94 | |
| 47. I tend to set specific goals/agendas for sessions | .54 | |
| Eigenvalue | 1.23 | |
| Total variance accounted (%) | 2.03 | |
| Items deleted due to insufficient factor loadings | | |
| 4. I incorporate parts of Triple P within another parenting or family intervention program | | |
| 13. I identify myself as a parenting practitioner | | |
| 35. I have difficulty accessing Triple P resource materials | | |
| 41. It is easy to incorporate Triple P into my job activities | | |
| 48. Triple P is frequently not appropriate for my client’s presenting problem | | |
| 50. Clients in my community often present with multiple vulnerabilities such as poverty, addiction issues, domestic violence, and mental health disorders | | |
| 51. I experience low availability of clients | | |
| Items deleted due to being unrelated to sustained program use | | |
| 20. I experience insufficient access to supervision | | |
| 29. It is difficult to me to coordinate with other practitioners involved with the families I work with | | |
| 30. Working with parents strongly interferes with my own family life | | |
| 46. I frequently experience lack of progress by children or families | | |
| 49. I frequently meet clients who need higher levels of Triple P than I am trained to provide | | |

*Note.* ^#^ Reverse coded items.

**Figure 1**

*Scree Plot for Principal Component Analysis*

*
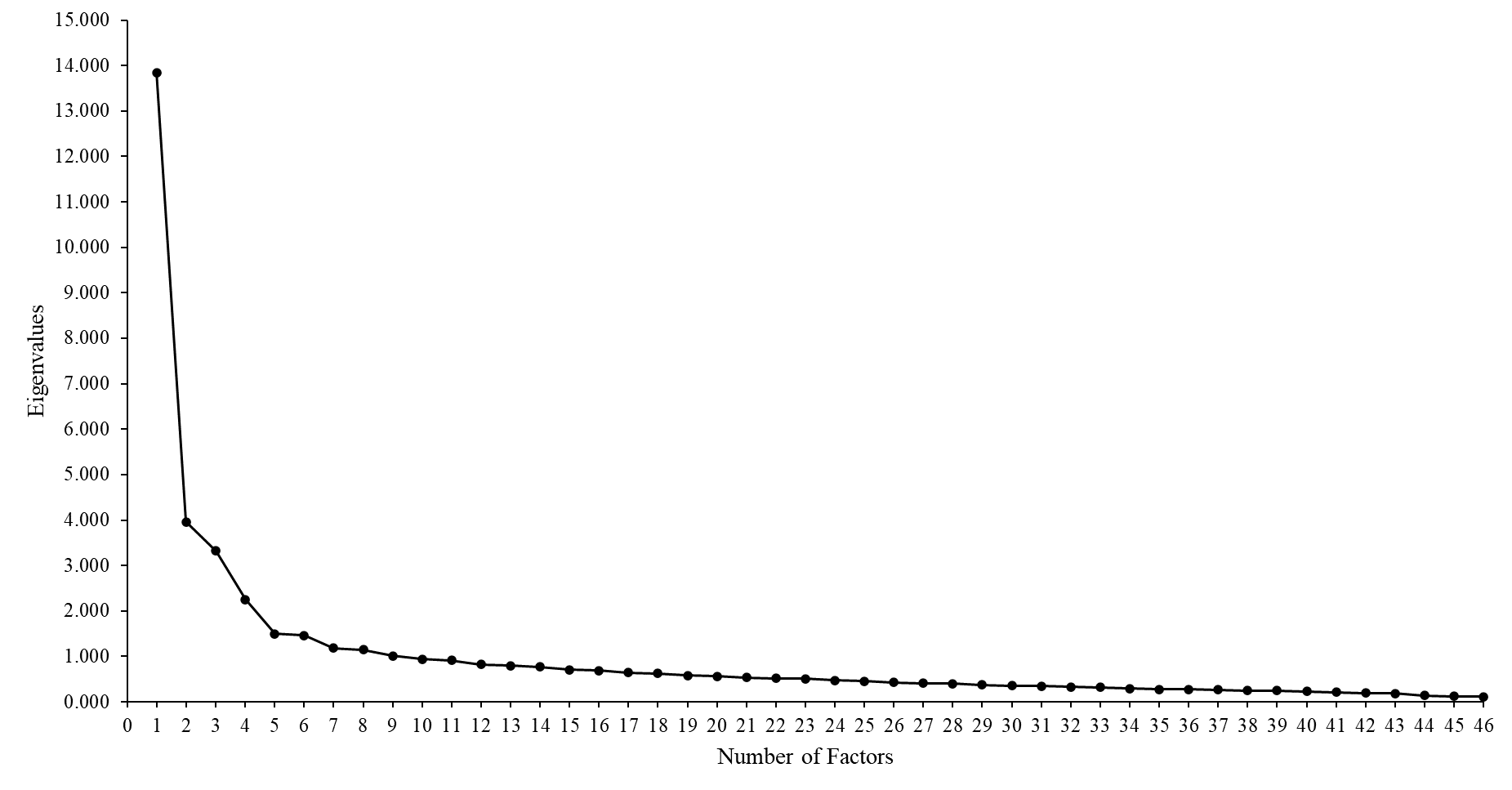
*

***Confirmatory Factor Analysis***

We conducted CFA in an independent sample (N = 599) to test the six-factor structure extracted from our EFA. Given that our data violated assumptions of normality, we used the robust maximum likelihood estimator (MLR) using *Mplus 7.0*, which is robust to non-normal observations (Beauducel & Herzberg, 2006; Muthen & Kaplan, 1992). As shown in *Table 3*, the initial model demonstrated acceptable fit according to RMSEA and SRMR, but chi-square, CFI suggested room for improvement. Investigating modification indices revealed that by allowing several error terms of the items correlated with each other could improve the model fit. Modifications were made one at a time until all model fit indexes met the acceptable level, which were three correlations between similar items, all of which had sound theoretical reasons. An adequate amount of data was explained by the final model (Model B); and the model fitted well, χ^2^(685) = 1828.80, *p* < .001, CFI = .900, SRMR = .057, RMSEA = .053 [90%CI: .050-.056]. A visualisation of the CFA results of the six-factor structure is displayed in *Figure 2*.

**Table 3**

*Confirmatory Factor Analysis for the Facilitators and Barriers Checklist*

| Model Number | χ^2^ | *df* | CFI | SRMR | RMSEA | RMSEA 90% CI |
| --- | --- | --- | --- | --- | --- | --- |
| Original Model | 2160.80^*^ | 687 | .871 | .056 | .060 | .057-.063 |
| A: Q22 with Q23 | 1952.80^*^ | 686 | .889 | .056 | .056 | .053-.058 |
| B: Q27 with Q28 | 1828.80^*^ | 685 | .900 | .057 | .053 | .050-.056 |

^*^ *p* < .001.

**Figure 2**

*Six-Factor Confirmatory Factor Analysis of the 39-Item Facilitators and Barriers Checklist with Three Error Covariances*


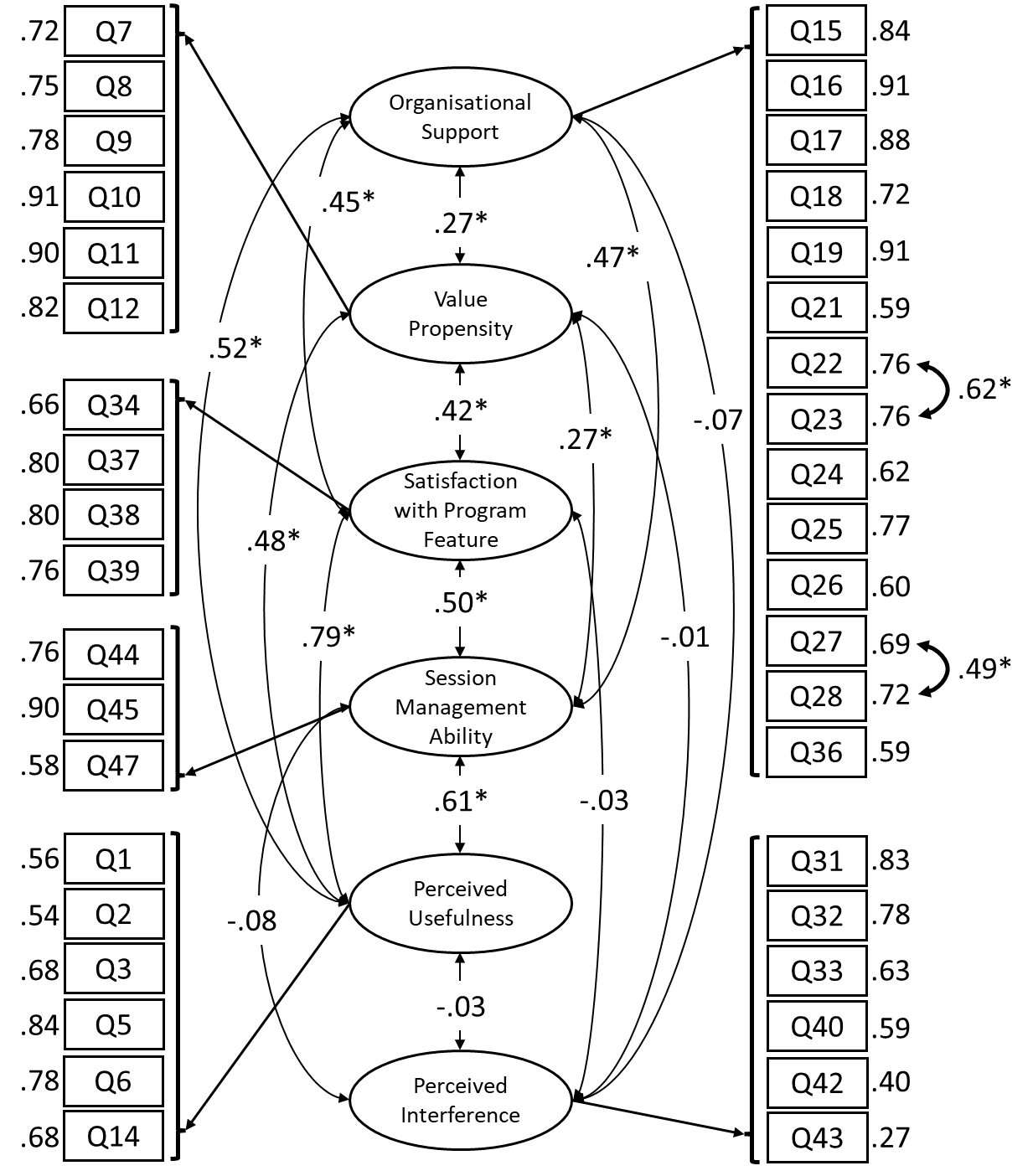


*Note.* All factor loadings are significant at *p* < .001 level.

^*^*p* < .001.

**Hypothesised Model**

**Figure 1**

*Hypothesised Model of Different Factors on the Sustained Use of the Triple P Program*

*
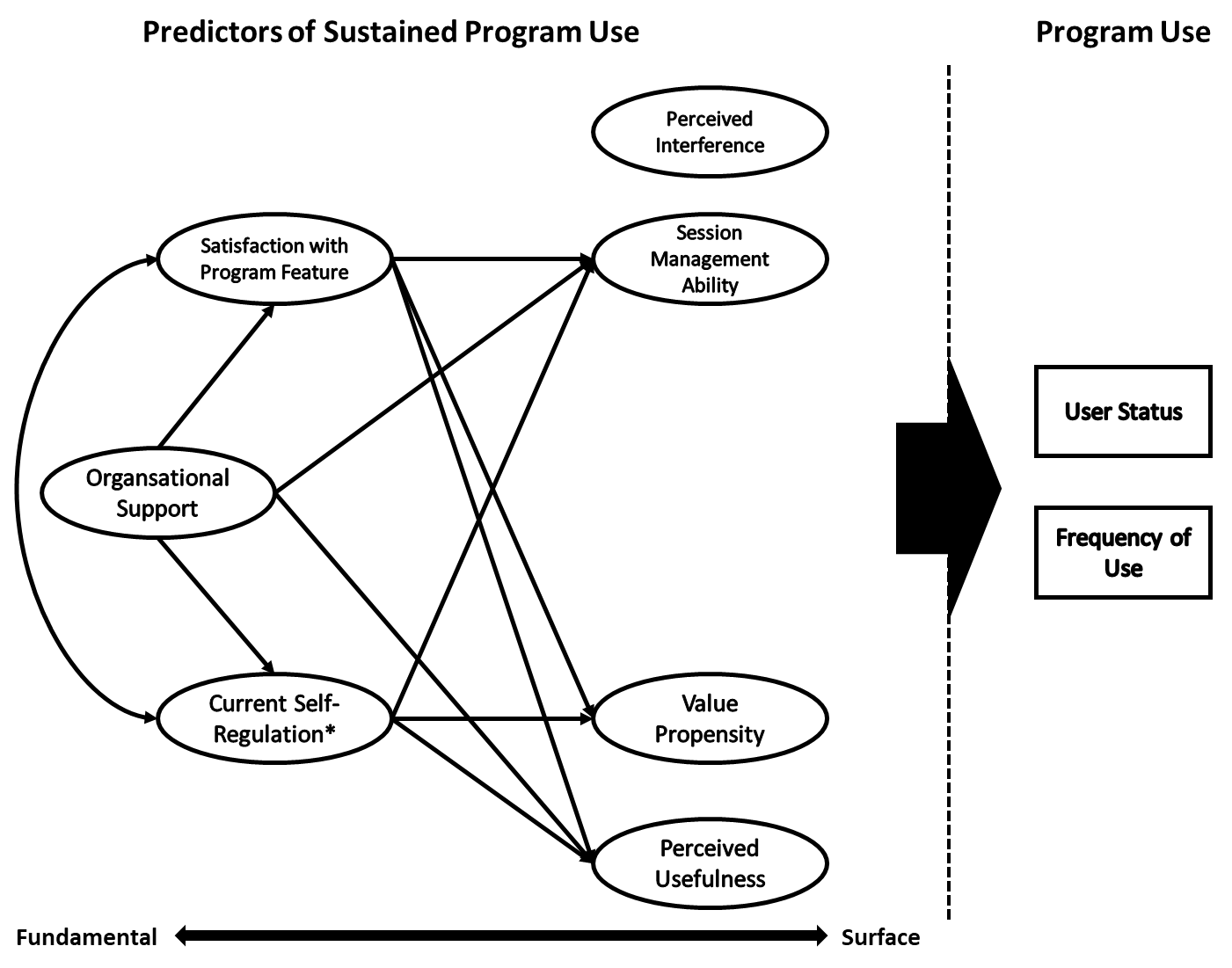
*

*Note.* All Factors are directly predicting program use. *Both PCSC and PSRS are combined to form the Current Self-Regulation factor.

**Measurement Model Evaluation**

**Table 4**

*Fit Indices for the Measurement Model (MLR)*

| Model Number | χ^2^ | *Df* | CFI | SRMR | RMSEA | RMSEA 90% CI |
| --- | --- | --- | --- | --- | --- | --- |
| Original Model | 8600.35^*^ | 1304 | .851 | .055 | .067 | .065-.068 |
| A: PCSC 1 with 2 | 7619.37^*^ | 1303 | .872 | .054 | .062 | .060-.063 |
| B: PSRS 2 with 3 | 6785.92^*^ | 1302 | .890 | .054 | .057 | .056-.059 |
| C: FBC 22 with 23 | 6249.41^*^ | 1301 | .901 | .054 | .054 | .053-.056 |

*Note.* PCSC: Practitioner Consultation Skill Checklist; PSRS: Parenting Self-Regulation Scale – Practitioner Version, FBC: Facilitators and Barriers Checklist.

^*^ *p* < .001.

References

Beauducel, A., & Herzberg, P. Y. (2006). On the performance of maximum likelihood versus means and variance adjusted weighted least squares estimation in CFA. *Structural Equation Modeling: A Multidisciplinary Journal, 13*(2), 186-203. <https://doi.org/10.1207/s15328007sem1302_2>

Conrad, Z., & Mario, R. S. (2014). Robust factor analysis in the presence of normality violations, missing data, and outliers: Empirical questions and possible solutions. *Tutorials in Quantitative Methods for Psychology, 10*(1), 40-55. <https://doi.org/10.20982/tqmp.10.1.p040>

Fabrigar, L. R., Wegener, D. T., MacCallum, R. C., & Strahan, E. J. (1999). Evaluating the use of exploratory factor analysis in psychological research. *Psychological Methods, 4*(3), 272-299. <https://doi.org/10.1037/1082-989X.4.3.272>

Field, A. P. (2018). *Discovering Statistics Using IBM SPSS Statistics* (5th ed.). SAGE Publications.

Gorsuch, R. L. (1983). *Factor analysis* (2nd ed.). Erlbaum.

MacCallum, R. C., Widaman, K. F., Zhang, S., & Hong, S. (1999). Sample size in factor analysis. *Psychological Methods, 4*(1), 84-99. <https://doi.org/10.1037/1082-989X.4.1.84>

Muthen, B., & Kaplan, D. (1992). A comparison of some methodologies for the factor analysis of non‐normal Likert variables: A note on the size of the model. *British Journal of Mathematical and Statistical Psychology, 45*(1), 19-30. <https://doi.org/10.1111/j.2044-8317.1992.tb00975.x>

West, S. G., Finch, J. F., & Curran, P. J. (1995). Structural equation models with nonnormal variables: Problems and remedies. In R. H. Hoyle (Ed.), *Structural equation modeling: Concepts, issues and applications* (pp. 56–75). Sage.
